# Supplementary material for: Unveiling hub genes and biological pathways: A bioinformatics analysis of Trauma-Induced Coagulopathy (TIC)
Source: PLoS One. 2025 Apr 29;20(4):e0322043. doi: 10.1371/journal.pone.0322043 (PMC12040245; doi:10.1371/journal.pone.0322043)
Supplement: Attachment — Submitted filename: PONE-D-24-36200.pdf [file pone.0322043.s001.pdf]

PLOS ONE

Unveiling Hub Genes and Biological Pathways: A Bioinformatics Analysis of Trauma-Induced Coagulopathy (TIC)

--Manuscript Draft--

Manuscript Number:	PONE-D-24-36200
Article Type:	Research Article
Full Title:	Unveiling Hub Genes and Biological Pathways: A Bioinformatics Analysis of Trauma-Induced Coagulopathy (TIC)
Short Title:	Identifying Key Genes and Pathways in Trauma-Induced Coagulopathy
Corresponding Author:	琳刚 张<br>Shanxi Medical University Eighth Hospital: Yuncheng Central Hospital<br>yuncheng city, CHINA
Keywords:	Biological Markers; Traumatic Coagulopathy; bioinformatics; Differentially Expressed Genes; Pathway Analysis; Protein-Protein Interaction Network; Hub Genes; ROC Analysis
Abstract:	Background<br><br>Trauma-Induced Coagulopathy is a severe condition that rapidly manifests following traumatic injury and is characterized by shock, hypoperfusion, and vascular damage. This study employed bioinformatics methods to identify crucial hub genes and pathways associated with TIC.<br><br>Methods<br><br>Microarray datasets (accession number GSE223245) were obtained from the Gene Expression Omnibus (GEO) database. The data were subjected analyses to identify the Differentially Expressed Genes (DEGs), which were further subjected to GO and KEGG pathway analyses. Subsequently, a Protein-Protein Interaction (PPI) network was constructed and hub DEGs closely linked to TIC were identified using CytoHubba, MCODE, and CTD scores. The diagnostic value of these hub genes was evaluated using Receiver Operating Characteristic (ROC) analysis.<br><br>Results<br><br>Among the analyzed genes, 269 were identified as DEGs, comprising 103 upregulated and 739 downregulated genes. Notably, several significant hub genes were associated with the development of TIC, as revealed by bioinformatic analyses.<br><br>Conclusions<br><br>This study highlights the critical impact of newly discovered genes on the development and progression of TIC. Further validation through experimental research and clinical trials is required to confirm these findings.<br><br>Ethical Compliance<br><br>All procedures performed in studies involving human participants were in accordance with the ethical standards of the institutional and/or national research committee and with the 1964 Helsinki Declaration and its later amendments or comparable ethical standards.
Order of Authors:	lin gang zhang
	Bo Li
	Jing Liu
	Yan Feng Bian
	Guo Xing Lin

Powered by Editorial Manager® and ProduXion Manager® from Aries Systems Corporation

	Ying Zhou
**Additional Information:**	
**Question**	**Response**
**Financial Disclosure**<br><br>Enter a financial disclosure statement that describes the sources of funding for the work included in this submission. Review the submission guidelines for detailed requirements. View published research articles from *PLOS ONE* for specific examples.<br><br>This statement is required for submission and **will appear in the published article** if the submission is accepted. Please make sure it is accurate.<br><br>**Funded studies**<br>Enter a statement with the following details:<br>• Initials of the authors who received each award<br>• Grant numbers awarded to each author<br>• The full name of each funder<br>• URL of each funder website<br>• Did the sponsors or funders play any role in the study design, data collection and analysis, decision to publish, or preparation of the manuscript?<br><br>Did you receive funding for this work?	Yes
Please add funding details.<br>as follow-up to "**Financial Disclosure**<br><br>Enter a financial disclosure statement that describes the sources of funding for the work included in this submission. Review the submission guidelines for detailed requirements. View published research articles from *PLOS ONE* for specific examples.<br><br>This statement is required for submission and **will appear in the published article** if the submission is accepted. Please make sure it is accurate.	2023 Yuncheng City Basic Research Program (Free Exploration Category) Projects<br>Item No：YCKJ-2023052

Powered by Editorial Manager® and ProduXion Manager® from Aries Systems Corporation

**Funded studies**<br>Enter a statement with the following details:<br>• Initials of the authors who received each award<br>• Grant numbers awarded to each author<br>• The full name of each funder<br>• URL of each funder website<br>• Did the sponsors or funders play any role in the study design, data collection and analysis, decision to publish, or preparation of the manuscript?<br><br>Did you receive funding for this work?"	
Please select the country of your main research funder (please select carefully as in some cases this is used in fee calculation).<br>as follow-up to "**Financial Disclosure**<br><br>Enter a financial disclosure statement that describes the sources of funding for the work included in this submission. Review the submission guidelines for detailed requirements. View published research articles from *PLOS ONE* for specific examples.<br><br>This statement is required for submission and **will appear in the published article** if the submission is accepted. Please make sure it is accurate.<br><br>**Funded studies**<br>Enter a statement with the following details:<br>• Initials of the authors who received each award<br>• Grant numbers awarded to each author<br>• The full name of each funder<br>• URL of each funder website<br>• Did the sponsors or funders play any role in the study design, data collection and analysis, decision to publish, or preparation of the manuscript?<br><br>Did you receive funding for this work?"	CHINA - CN
**Competing Interests**	The authors have declared that no competing interests exist.

Use the instructions below to enter a competing interest statement for this submission. On behalf of all authors, disclose any competing interests that could be perceived to bias this work—acknowledging all financial support and any other relevant financial or non-financial competing interests.<br><br>This statement is required for submission and **will appear in the published article** if the submission is accepted. Please make sure it is accurate and that any funding sources listed in your Funding Information later in the submission form are also declared in your Financial Disclosure statement.<br><br>View published research articles from *PLOS ONE* for specific examples.<br><br>**NO authors have competing interests**<br><br>Enter: *The authors have declared that no competing interests exist.*<br><br>**Authors with competing interests**<br><br>Enter competing interest details beginning with this statement:<br><br>*I have read the journal's policy and the authors of this manuscript have the following competing interests: [insert competing interests here]*<br><br>* typeset	
**Ethics Statement**<br><br>Enter an ethics statement for this submission. This statement is required if the study involved:<br><br>• Human participants<br>• Human specimens or tissue<br>• Vertebrate animals or cephalopods<br>• Vertebrate embryos or tissues	N/A

Powered by Editorial Manager® and ProduXion Manager® from Aries Systems Corporation

• Field research<br><br>Write "N/A" if the submission does not require an ethics statement.<br><br>General guidance is provided below. Consult the submission guidelines for detailed instructions. **Make sure that all information entered here is included in the Methods section of the manuscript.**	

**Format for specific study types**<br><br>**Human Subject Research (involving human participants and/or tissue)**<br>• Give the name of the institutional review board or ethics committee that approved the study<br>• Include the approval number and/or a statement indicating approval of this research<br>• Indicate the form of consent obtained (written/oral) or the reason that consent was not obtained (e.g. the data were analyzed anonymously)<br><br>**Animal Research (involving vertebrate animals, embryos or tissues)**<br>• Provide the name of the Institutional Animal Care and Use Committee (IACUC) or other relevant ethics board that reviewed the study protocol, and indicate whether they approved this research or granted a formal waiver of ethical approval<br>• Include an approval number if one was obtained<br>• If the study involved *non-human primates*, add *additional details* about animal welfare and steps taken to ameliorate suffering<br>• If anesthesia, euthanasia, or any kind of animal sacrifice is part of the study, include briefly which substances and/or methods were applied<br><br>**Field Research**<br><br>Include the following details if this study involves the collection of plant, animal, or other materials from a natural setting:<br>• Field permit number<br>• Name of the institution or relevant body that granted permission	
**Data Availability**<br><br>Authors are required to make all data underlying the findings described fully available, without restriction, and from the time of publication. PLOS allows rare exceptions to address legal and ethical concerns. See the PLOS Data Policy and FAQ for detailed information.	Yes - all data are fully available without restriction

Powered by Editorial Manager® and ProduXion Manager® from Aries Systems Corporation

A Data Availability Statement describing where the data can be found is required at submission. Your answers to this question constitute the Data Availability Statement and **will be published in the article**, if accepted.<br><br>**Important:** Stating 'data available on request from the author' is not sufficient. If your data are only available upon request, select 'No' for the first question and explain your exceptional situation in the text box.<br><br>Do the authors confirm that all data underlying the findings described in their manuscript are fully available without restriction?	
**Describe where the data may be found in full sentences. If you are copying our sample text, replace any instances of XXX with the appropriate details.**<br><br>• If the data are **held or will be held in a public repository**, include URLs, accession numbers or DOIs. If this information will only be available after acceptance, indicate this by ticking the box below. For example: *All XXX files are available from the XXX database (accession number(s) XXX, XXX.).*<br>• If the data are all contained **within the manuscript and/or Supporting Information files**, enter the following: *All relevant data are within the manuscript and its Supporting Information files.*<br>• If neither of these applies but you are able to provide **details of access elsewhere**, with or without limitations, please do so. For example:<br><br>*Data cannot be shared publicly because of [XXX]. Data are available from the XXX Institutional Data Access / Ethics Committee (contact via XXX) for researchers who meet the criteria for access to confidential data.*<br><br>*The data underlying the results presented in the study are available from (include the name of the third party*	All files are available from the GEO database

Powered by Editorial Manager® and ProduXion Manager® from Aries Systems Corporation

and contact information or URL).<br>• This text is appropriate if the data are owned by a third party and authors do not have permission to share the data.<br><br>* typeset	
Additional data availability information:	Tick here if the URLs/accession numbers/DOIs will be available only after acceptance of the manuscript for publication so that we can ensure their inclusion before publication.

Powered by Editorial Manager® and ProduXion Manager® from Aries Systems Corporation

Manuscript

Click here to access/download;Manuscript;renamed_c34e3.docx

Unveiling Hub Genes and Biological Pathways: A Bioinformatics Analysis of Trauma-Induced Coagulopathy (TIC)

Lin Gang Zhang[1*¶], Bo Li[2¶] Jing Liu[3] Yan Feng Bian[4] Guo Xing Lin[5] Ying Zhou[1]

1Emergency Department, Yuncheng Central Hospital affiliated to Shanxi Medical University,Yuncheng, Shanxi, China;
2 Reproductive Medicine Department, Yuncheng Central Hospital affiliated to Shanxi Medical University, Yuncheng, Shanxi, China; 315426935@qq.com
3 Pathology Department,Yuncheng Central Hospital affiliated to Shanxi Medical University,Yuncheng, Shanxi, China; liujing0420@126.com
4 Emergency sungery,Shanxi Bethune Hospital,Shanxi Academy of Medical Sciences,Tongji Shanxi Hospital,Third Hospital of Shanxi Medical University,China; 659288625@qq.com
5 Emergency Department, Hebei province Xingtai Third People's Hospital,China; 2361013627@qq.com
* Corresponding author
E-mail:zhanglinggang@163.com
¶ Lingang Zhang and Bo Li contributed equally to this article.

Abstract

Background: Trauma-Induced Coagulopathy is a severe condition that rapidly manifests following traumatic injury and is characterized by shock, hypoperfusion, and vascular damage. This study employed bioinformatics methods to identify crucial hub genes and pathways associated with TIC.

Methods: Microarray datasets (accession number GSE223245) were obtained from the Gene Expression Omnibus (GEO) database. The data were subjected analyses to identify the Differentially Expressed Genes (DEGs), which were further subjected to GO and KEGG pathway analyses. Subsequently, a Protein-Protein Interaction (PPI) network was constructed and hub DEGs closely linked to TIC were identified using CytoHubba, MCODE, and CTD scores. The diagnostic value of these hub genes was evaluated using Receiver Operating Characteristic (ROC) analysis.

Results: Among the analyzed genes, 269 were identified as DEGs, comprising 103 upregulated and 739 downregulated genes. Notably, several significant hub genes were associated with the development of TIC, as revealed by bioinformatic analyses.

Conclusions: This study highlights the critical impact of newly discovered genes on the development and progression of TIC. Further validation through experimental research and clinical trials is required to confirm these findings.
Ethical Compliance: All procedures performed in studies involving human participants were in accordance with the ethical standards of the institutional and/or national research committee and with the 1964 Helsinki Declaration and its later amendments or comparable ethical standards.
Data Access Statement: Research data supporting this publication are available from the GEO database.

Keywords: Biological Markers, Traumatic Coagulopathy, Bioinformatics, Differentially Expressed Genes, Pathway Analysis, Protein-Protein Interaction Network, Hub Genes, ROC Analysis.

1. Introduction

Uncontrolled hemorrhage is a significant preventable factor that contributes to mortality in patients with traumatic injuries. Additionally, among individuals under the age of 50 years, injury is the second most significant cause of mortality, closely following infectious diseases [1]. Impaired coagulation following sudden death due to injury has been recognized and recorded for centuries [2]. TIC refers to the abnormal coagulation processes that occur as a result of trauma. Severe trauma can result in the development of TIC through various mechanisms, including activation of protein C, disruption of the endothelial glycocalyx, consumption of fibrinogen, and platelet dysfunction. The ultimate objective of personalized medicine for patients at risk of TIC is to ensure the delivery of the most suitable products to each individual patient at the right time. However, despite significant research efforts, our current understanding of the pathophysiology of TIC remains incomplete. This incompleteness is further compounded by limitations in diagnostic testing, which contributes to the imprecision of current clinical decisions.

The rapid development of innovative technologies, including next-generation sequencing (NGS), has significantly accelerated the exploration of diagnostic and therapeutic biomarkers for TIC. Bioinformatics analysis plays a crucial role in uncovering novel clues and essential data for the identification of reliable and functional differentially expressed genes (DEGs) and non-coding transcripts [3]. Furthermore, integrated studies that combine data from various medical sources not only save resources, but also provide valuable evidence for mapping the molecular pathogenesis networks of diseases.

In this study, we assessed the gene expression profile for traumatic coagulopathy from the GEO database. The GEO database is an open-access resource that offers comprehensive genetic information, making it a valuable tool for bioinformatic analysis and identification of new disease targets [4]. Using bioinformatics methods, we successfully identified differentially expressed genes (DEGs). DEGs were subsequently subjected to analysis using PPI, GO, KEGG, and pathways. Furthermore, the study analyzed the pathways associated with TIC, as well as the interactions between DEGs and pathways. Subsequently, The hub DEGs were identified using CytoHubba, MCODE, and CTD scores.Lastly, ROC analysis was conducted to evaluate the

diagnostic value of the identified hub genes.As a result, the identified hub genes have the potential to become a novel area of research focus. The elucidated molecular mechanisms and signaling pathways may provide valuable insights into understanding TIC.

2.Methods

Microarray data retrieval

Coagulopathy datasets were acquired from the National Center for Biotechnology Information (NCBI) GEO (http://www.ncbi.nlm.nih.gov/geo)[5] public repository. Lastly, we obtained GSE223245 from the NCBI GEO. The GSE223245 dataset was generated using the GPL33038 platform, which includes ceRNA chipset samples from Homo sapiens. This dataset comprised 12 patients with traumatic brain injury (TBI) and four healthy controls, with peripheral blood mononuclear cells (PBMC) collected for analysis.

Data Processing and Differentially Expressed Genes Identification

Microarray data were accessed from GEO using the R package "GEOquery.” Differentially expressed genes (DEGs) were obtained from the microarray data using the R package "limma.” All identified differentially expressed genes (DEGs) met the criteria of p-value < 0.05 and log2 (fold-change) ≥ 1. The resulting differentially expressed genes (DEGs) were visualized using a Volcano Plot created using the R packages "ggplot2"[6] and "dplyr.” Additionally, a Heatmap was generated using the R package "pheatmap" to further visualize DEGs.

Functional enrichment analysis

Gene Set Enrichment Analysis (GSEA) [7] was performed using the R package "clusterProfiler"[8]. The process of conducting Gene Ontology (GO) and Kyoto Encyclopedia of Genes and Genomes (KEGG) pathway enrichment analyses of Differentially Expressed Genes (DEGs) were performed using the R package "clusterProfiler"[8]. The results were visualized using the R packages "ggplot2"[6].

Analysis of protein–protein interactions (PPI) and identifcation of Hub genes

Protein-protein interaction network construction and module analysis were performed following established protocols, as described in previous studies [9]. The overlapping DEGs were subjected to protein-protein interaction (PPI) analysis using the STRING database(https://string-db.org/)[10]. Network visualization of the resulting interactions was achieved using Cytoscape version 3.8.2[11]. Hub genes were identified using CytoHubba and MCODE plugins, which were implemented in Cytoscape version 3.8.2.

Expression and ROC Analysis

Based on their centrality values, the top ten genes in the protein-protein interaction (PPI) network were identified as critical genes. Receiver operating characteristic (ROC) curves were generated

using the pROC R package to analyze the performance of the classification model, and ROC curves were used to assess the predictive capability of the identified biomarkers.

3.Results

3.1 Identification of Differentially Expressed Genes

A flowchart depicting the overall data-screening strategy is shown in Fig.1. In the coagulopathy dataset GSE223245, 823 DEGs were filtered when we compared the 12 coagulopathy samples with 4 healthy controls In the GSE223245 dataset, the differential analysis revealed a total of 269 differentially expressed genes (DEGs), with 103 genes up-regulated and 166 genes down-regulated in coagulopathy samples compared to healthy samples.The DEGs were visualized through Volcano Plots, Heatmaps, PCA, and Boxplots, providing comprehensive insights into their expression patterns. (fig.2 A-D). We observed a correlation between the DEGs (fig.3 a,b)

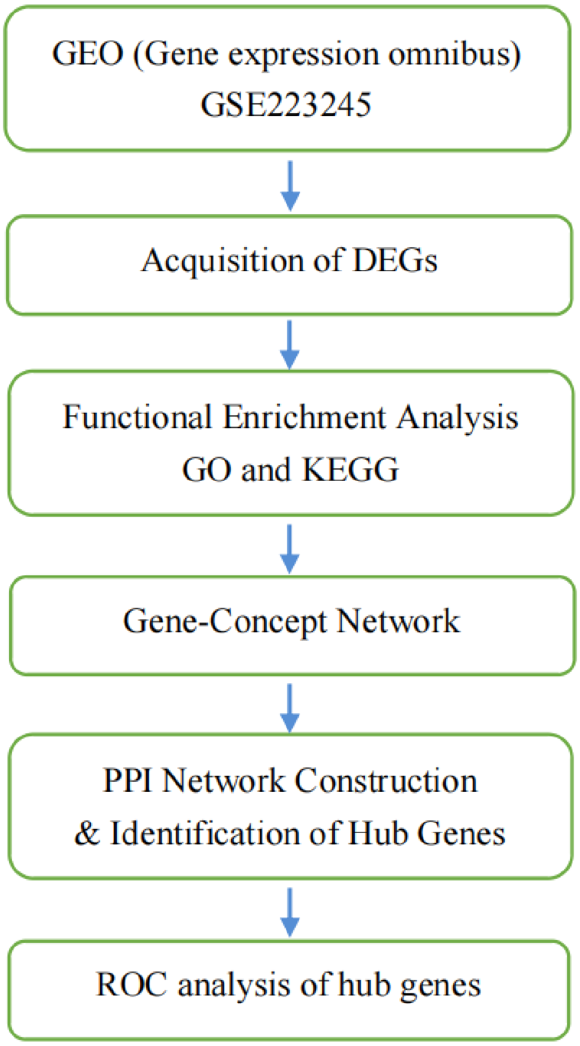

Fig1: The multistep screening strategy for bioinformatics data is presented in the flowchart below.

3.2 GO and KEGG enrichment pathway analysis

To explore the functional aspects of the DEGs more comprehensively, gene symbols were analyzed using the latest versions of the GO and KEGG pathway databases. This analysis aimed to ascertain the potential functions associated with DEGs.

The results of the KEGG analysis demonstrated significant enrichment primarily in some pathways: tryptophan metabolism, autoimmune thyroid disease, steroid hormone biosynthesis, inflammatory bowel disease, natural killer cell-mediated cytotoxicity, viral protein interaction with cytoking and cytoking receptors, Pantothenate and CoA biosynthesis, and the NOD-like receptor signaling pathway(fig.4 a). GO analysis indicated that the DEGs were primarily

enriched in some categories: regulation of immune effector process, regulation of response to biotic stimulus, regulation of cell killing, regulation of innate immune response, and defense response to virus (fig.4 b-c). These pathways play a significant role in the occurrence and progression of traumatic coagulopathy.

A correlation between the primary pathways and genes(fig.5 a-b,fig6 b). These pathways primarily exhibit a high concentration in defense responses to viruses, regulation of cell killing, regulation of immune effector processes, regulation of innate immune responses, and regulation of responses to biotic stimuli. The genes intricately linked to these pathways are CXCL6,CD160,KLRC4,DDX60,IFIT1,RSAD2,CFH,VSIG4.It is possible to gain an understanding of the interrelationships between these pathways(fig.6 a). The pathways that exhibit the highest concentration are predominantly those regulating cell killing, regulation of immune effector processes, regulation of innate immune response, and regulation of response to biotic stimulus.

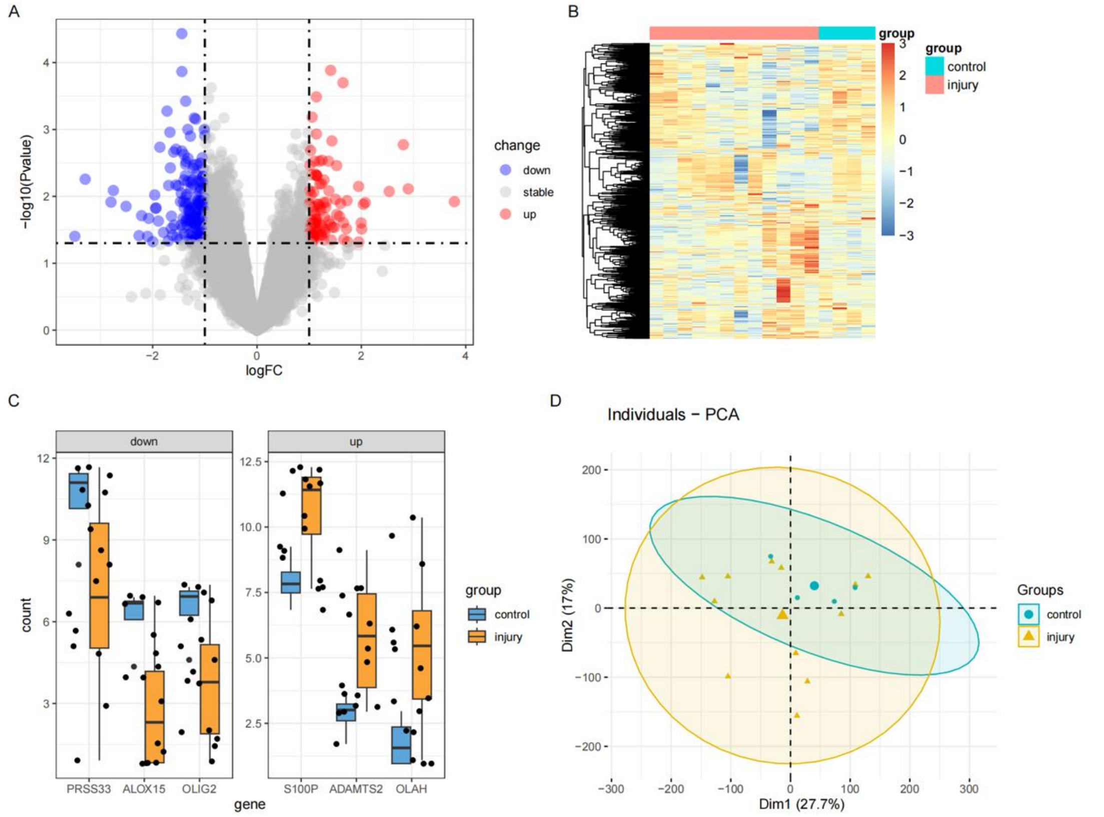

Fig.2 DEG in traumatic coagulopathy. **A** Volcano plot of DEGs in GSE223245; **B** Clustered heatmap of DEGs in GSE223245; **C** The box plot showcases the expression levels of the first three up-regulated genes and the last three down-regulated genes; **D** the PCA score plots show a comparison between the coagulopathy group and the healthy group in the datasets.

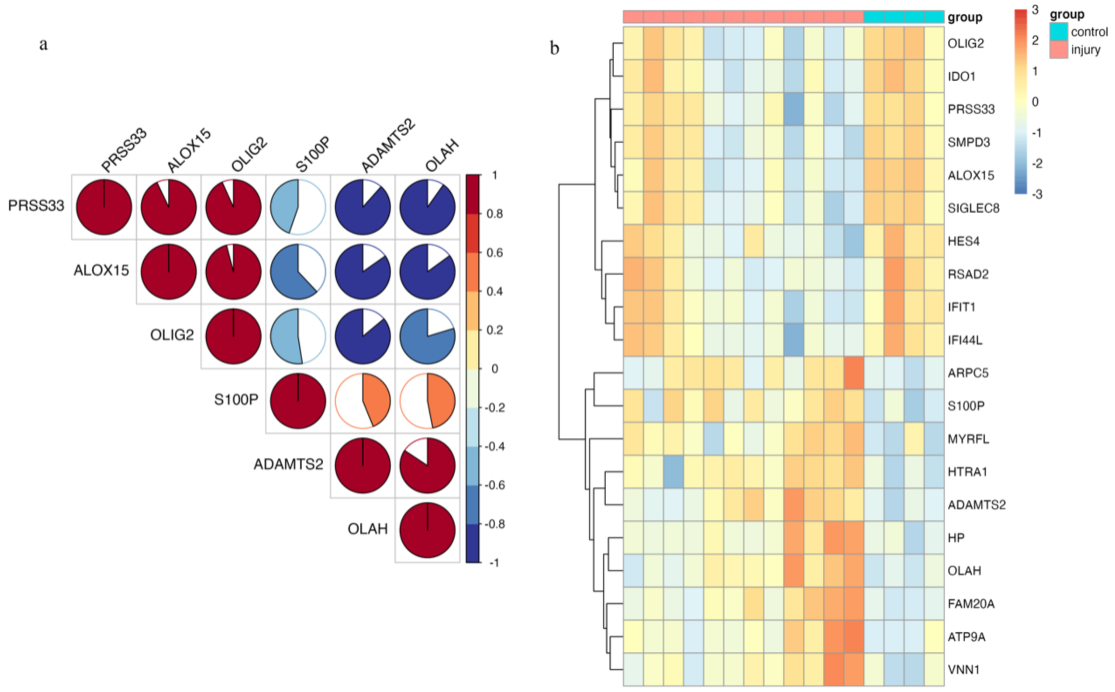

Fig.3 (a) The corrplot showcases the expression levels of the first three up-regulated genes and the last three down-regulated genes. (b) The Clustered heatmap showcases the expression levels of the first ten up-regulated genes and the last ten down-regulated genes.

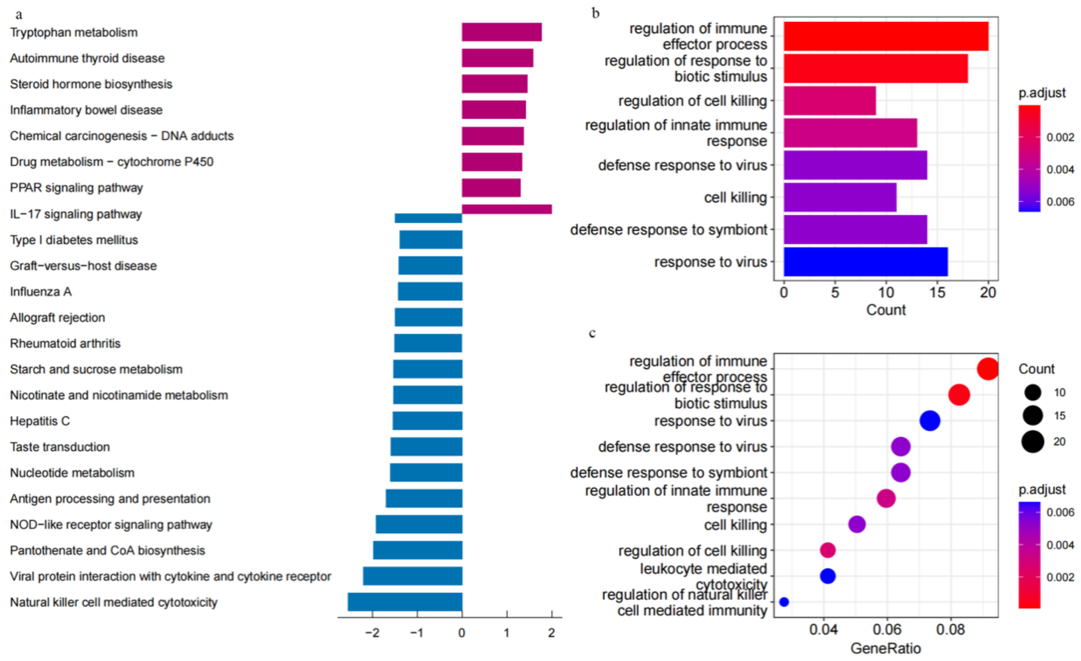

- Fig.4 Enrichment analysis of the DEGs. (a) Outcomes of the KEGG enrichment analysis for the DEGs.(b and c) Outcomes of the GO functional analysis demonstrating the significantly enriched terms for the DEGs.

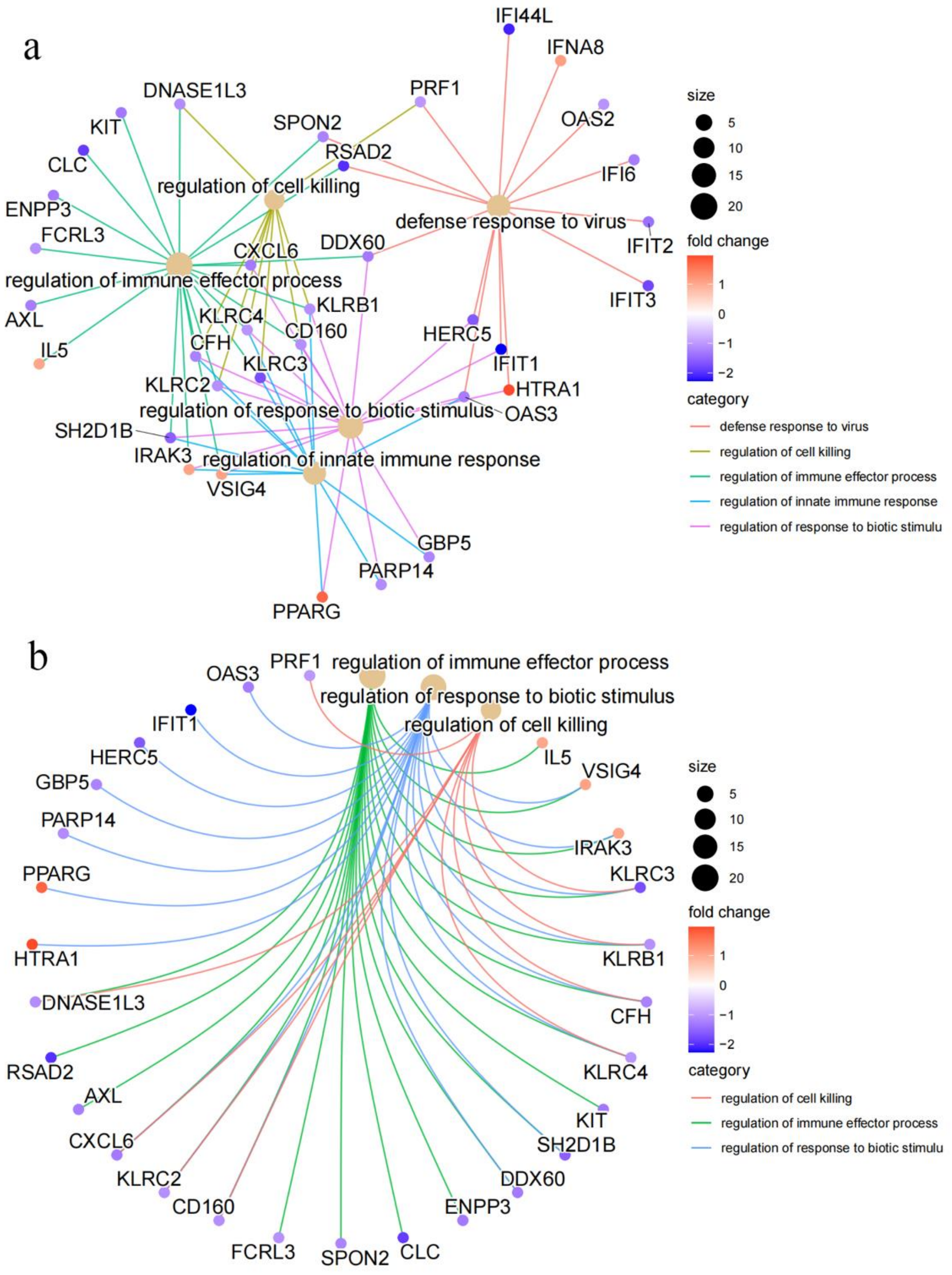

Fig.5 the relationship between the main pathways and DEGs

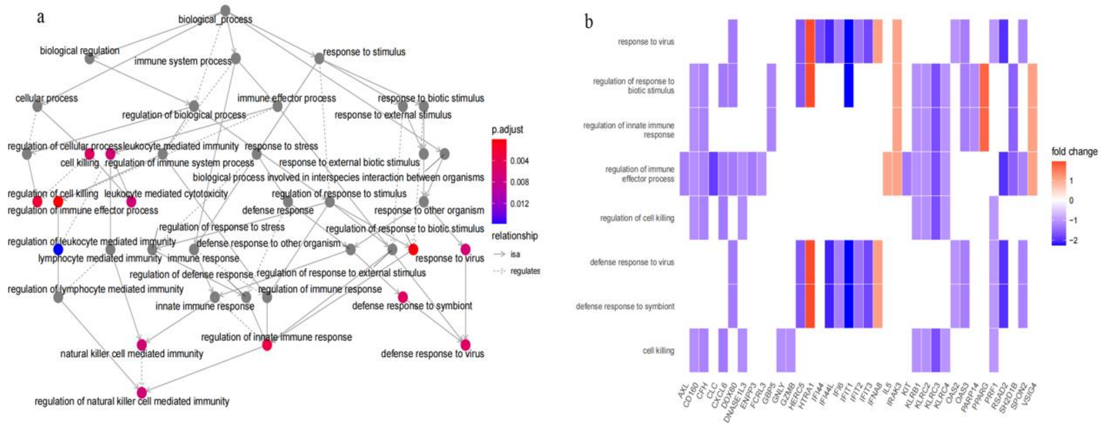

Fig.6 There is a correlation between different pathways (a).The Clustered heatmap showcases the relationship between the main pathways and DEGs (b).

3.3 Exploring PPI Networks and Identifying Hub Genes: Uncovering Key Interactions in DEGs

A protein-protein interaction (PPI) network was constructed using the PPI pairs from the STRING database, representing the interactions among proteins encoded by the DEGs. The PPI network was visualized using Cytoscape, allowing for a comprehensive analysis of protein interactions (fig.7 a). Significant modules (gene clusters) were identified using the MCODE plugin, which facilitates the detection of densely connected regions within the PPI network.

The PPI network was analyzed using the MCC algorithm from the CytoHubba plugin, resulting in the identification of 10 hub genes as the top candidates. OAS2, OAS3, IFIT2, IFIT1, IFIT3, HERC5, IFI44, IFI44L, RSAD2, and DDX60 (fig.7 b).

3.4 Assessing the Diagnostic Value of Hub Genes

To validate the diagnostic value of the 10 hub genes obtained from the previous analysis, ROC curves were constructed and the corresponding area under the curve (AUC) was calculated for gene expression levels in the traumatic coagulopathy datasets(fig.8). The AUC for OAS2, OAS3, IFIT2, IFIT1, IFIT3, HERC5, IFI44, IFI44L, RSAD2, DDX60 were 0.854,0.833, 0.854, 0.854, 0.854,0.812,0.833,0.833,0.833,0.875.

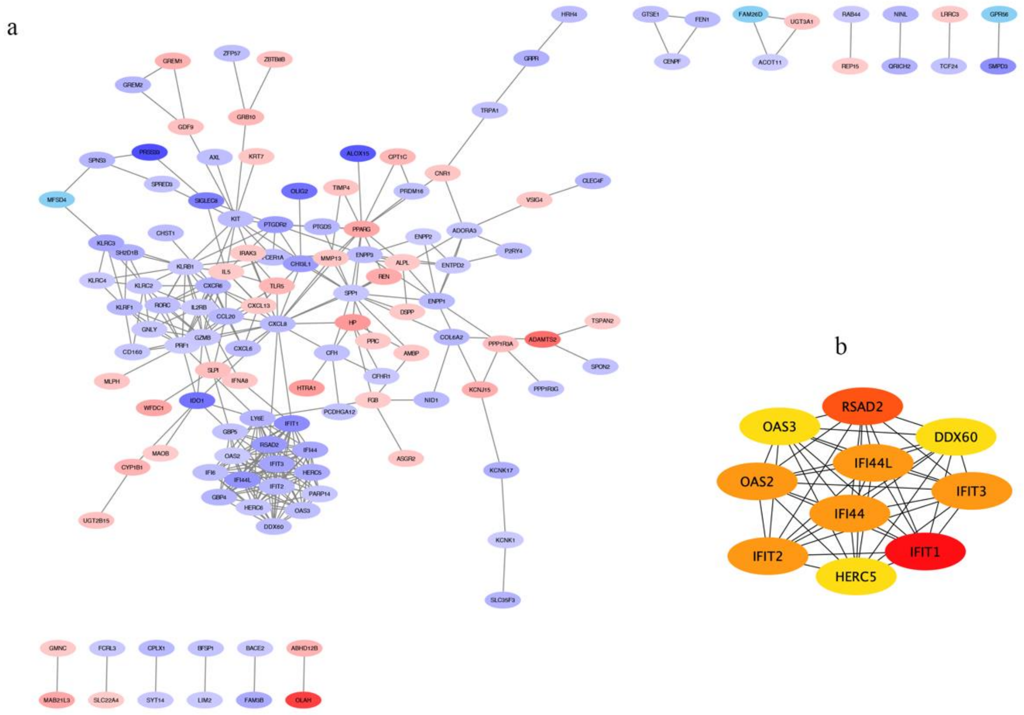

Fig.7 PPI network construction (a); Top 10 hub genes explored by CytoHubba. (b)

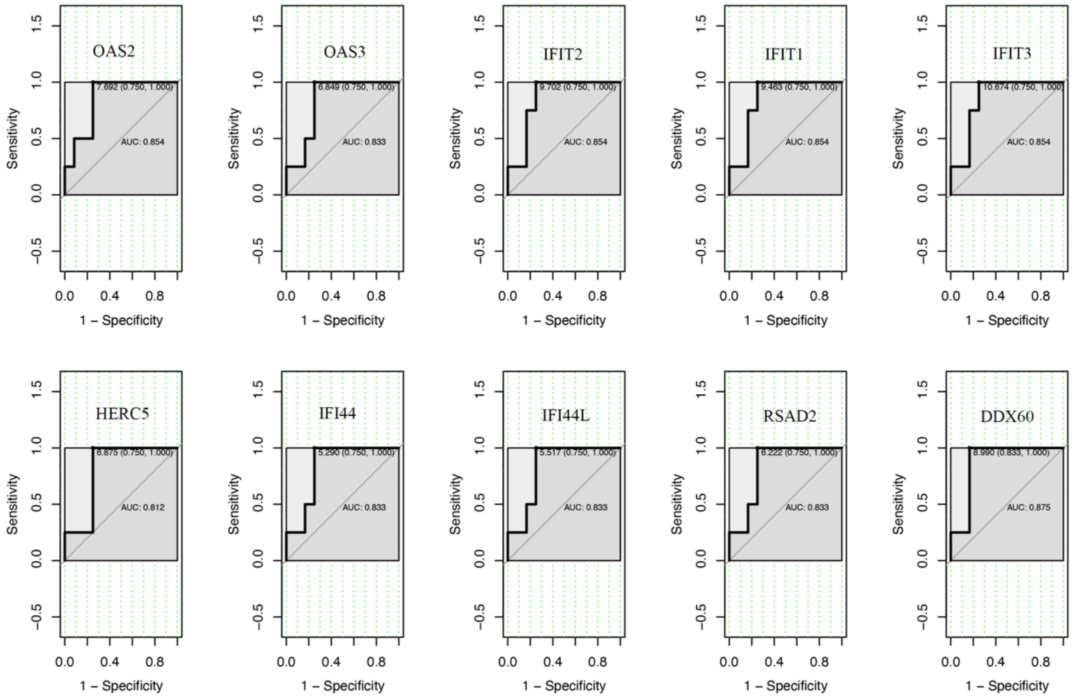

Fig.8 ROC curves were employed to evaluate the diagnostic utility of the 10 hub genes in traumatic coagulopathy.

4. Discussion

In the global context, injuries hold the position of being the fourth leading cause of mortality [1].In civilian [12] and military [13] settings, early preventable deaths after injury are mainly caused by uncontrolled hemorrhage[12-17], whereas later preventable deaths are typically

attributed to hypercoagulability [18]. After experiencing massive trauma with the presence of shock, hypoperfusion, and vascular damage, Trauma-Induced Coagulopathy (TIC) develops rapidly. This condition impairs the ability of the body to form blood clots and can lead to increased bleeding risk[19]. A comprehensive understanding of TIC pathophysiology is indispensable for lowering trauma-related mortality rates [20]. The mechanisms underlying TIC involve the activation of protein C, disruption of the endothelial glycocalyx, decreased fibrinogen levels, and impaired platelet function [19]. Nevertheless, the pathogenesis of TIC remains elusive and there is a scarcity of effective therapeutic strategies to address this condition. In this context, it is imperative to enhance our understanding of TIC pathogenesis and actively seek potential therapeutic targets. Using various bioinformatic methods, the current study successfully retrieved DEGs from TIC-related microarray datasets sourced from the GEO database. Furthermore, the study encompassed GO and KEGG pathway enrichment analyses. Subsequently, a PPI network was assembled to identify the top 10 hub genes among the DEGs. Ten hub genes (OAS2, OAS3, IFIT2, IFIT1, IFIT3, HERC5, IFI44, IFI44L, RSAD2, and DDX60) were selected to validate their diagnostic value in patients with TIC ($P<0.05$). These genes possess significant potential for predicting the risk of TIC, making them crucial candidates for further investigation.

Multiple hypotheses have been proposed to explain the underlying mechanisms driving the process, suggesting that tissue injury and shock work synergistically to activate the endothelium, platelets, and immune system. This activation leads to the production of various mediators that have the combined effects of reducing fibrinogen levels, impairing platelet function, and compromising thrombin generation. Consequently, these processes ultimately result in inadequate clot formation, leading to compromised hemostasis. During viral infections, similar to bacterial infections, the coagulation system undergoes activation, and in the initial stages,the activation of the coagulation cascade could potentially serve as a host defense mechanism, working to impede the spread of viruses [21]. Type I interferons (IFNs) play a crucial role in shaping both innate and adaptive immune responses. Activation of the Janus kinase (JAK)-signal transducer and activator of transcription (STAT) pathway through type I IFN signaling leads to the transcription of IFN-stimulated genes (ISGs) [22].

The study identified RSAD2, IFIT1, IFIT2, IFIT3, OAS2, OAS3, IFI44, and IFI44L as the eight hub genes within the ISGs (IFN-stimulated genes). RSAD2, also known as Radical S-adenosyl methionine domain-containing 2, is an interferon-stimulated gene that is significantly upregulated upon viral infection. It responds to both type I and type II interferon signaling through the JAK/STAT pathway [23]. Previous studies have demonstrated that RSAD2 exhibits broad antiviral activity against multiple enveloped viruses. Its function as an antiviral agent has been observed in various viral infections, highlighting its potential as a promising therapeutic target for combating enveloped viruses [24]. By inhibiting the NF-κB pathway, the suppression of RASD2 can effectively decrease the viability of CD19+ B cells and enhance their apoptosis. Furthermore, silencing of RASD2 leads to a reduction in the expression of IL-10[25].

The IFN-induced proteins with tetratricopeptide repeats (IFITs) family is one of the numerous IFN-stimulated gene families. Within this family, there was a cluster of duplicated loci. IFIT1, IFIT2, IFIT3, and IFIT5 are present in most mammals [26]. Besides initiating a cytokine storm [27], SARS-CoV-2 infection leads to activation of the coagulation pathway by causing damage to vascular endothelial cells [28]. The presence of SARS-CoV-2 suggests potential protective effects of IFIT1, IFIT2, and IFIT3 expression in gingival epithelial cells (GECs) against

coronavirus infection [29]. Consequently, the expression of the IFITs family may exert an inhibitory effect on the activation of the coagulation pathway.The 2'-5'-oligoadenylate synthetases (OAS), including OAS1, OAS2, and OAS3, are classified as interferon-induced genes that have long been associated with an antiviral function [30]. Their downstream products can trigger the activation of RNase L, an enzyme that facilitates the breakdown of both cellular and viral components [31]. The IFI44 gene family is recognized as a newly diversified mediator of immune responses in oysters [32].

DDX60, a novel DEAD-box RNA helicase, is an upstream regulator of RIG-I in the innate immune response. It was first discovered through microarray research focused on genes induced by measles virus infection in dendritic cells (DCs) [33]. We present experimental findings that support the co-localization of DDX60 with the RIG-I protein, RIG-I ligand, and a stress granule marker, G3BP. This colocalization provides strong evidence that DDX60 plays a role in the recognition of viral RNA by RIG-I [34]. In this study, we discovered that DDX60 is involved in multiple signaling pathways related to immune regulation. Specifically, DDX60 is implicated in the "regulation of immune effector process," "regulation of response to biotic stimulus," and "defense response to virus" signaling pathways (fig 5a). These findings highlight the significance of DDX60 in orchestrating immune responses against viral infection.

HECT and RCC1-containing protein 5 (HERC5) are immune proteins with potent antiviral properties. It is specifically induced in response to IFN-α/β signal transduction, which plays a crucial role in the innate immune response against viral infections [35]. HERC5 demonstrates antiviral efficacy against a wide range of divergent viruses, including retroviruses such as Human Immunodeficiency Virus (HIV) and Simian Immunodeficiency Virus (SIV), as well as papillomaviruses and influenza viruses. Its ability to combat these diverse viral pathogens underscores its broad-spectrum antiviral function of HERC5 [36] [37]. In our study, we revealed the involvement of HERC5 in key signaling pathways associated with immune regulation. Specifically, we found that HERC5 plays a significant role in the "regulation of response to biotic stimulus" and "defense response to virus" signaling pathways(fig 5a). These findings underscore the importance of HERC5 in modulating immune responses to various biotic stimuli, including viral infection.

This study has certain limitations that should be considered. First, one of the main limitations of this study was the lack of clinical data. Furthermore, despite performing a comprehensive bioinformatics analysis in the present study, we regrettably did not proceed with additional experiments. Hence, it is imperative to further investigate the specific mechanisms underlying TIC through in vivo and in vitro experiments.

5. Conclusions

In summary, through an extensive bioinformatics analysis, we successfully identified the DEGs. We made a groundbreaking discovery by uncovering the previously unknown crosstalk between genes and pathways associated with TIC.We, unearthed ten hub genes, and substantiated their diagnostic value by utilizing ROC curves. These ten hub genes are OAS2, OAS3, IFIT2, IFIT1, IFIT3, HERC5, IFI44, IFI44L, RSAD2, DDX60.

These eight genes are classified as Interferon-Stimulated Genes (ISGs), namely, RSAD2, IFIT1, IFIT2, IFIT3, OAS2, OAS3, IFI44 and IFI44L.All of these genes are involved in the immune system and are relevant to antiviral defense. Consequently, they influence the coagulation system.

Our findings indicate that HERC5 plays a crucial role in the signaling pathways related to the "regulation of response to biotic stimulus" and "defense response to viruses." DDX60 is implicated in the "regulation of immune effector process," "regulation of response to biotic stimulus," and "defense response to virus" signaling pathways

Acknowledgments

Ethical Approval

The data for this study were obtained from the public GEO database, and ethical committee approval was not required.

Competing interests

The authors declare no conflicts of interest regarding the publication of this paper.

References

1. Moore EE, Moore HB, Kornblith LZ, Neal MD, Hoffman M, Mutch NJ, et al. Trauma-induced coagulopathy. Nat Rev Dis Primers. 2021;7(1):30. Epub 2021/05/01. doi: 10.1038/s41572-021-00264-3. PubMed PMID: 33927200; PubMed Central PMCID: PMCPMC9107773.
2. Macfarlane RG, Biggs R. Fibrinolysis; its mechanism and significance. Blood. 1948;3(10):1167-87. Epub 1948/10/01. PubMed PMID: 18884682.
3. Vernon ST, Hansen T, Kott KA, Yang JY, O'Sullivan JF, Figtree GA. Utilizing state-of-the-art "omics" technology and bioinformatics to identify new biological mechanisms and biomarkers for coronary artery disease. Microcirculation. 2019;26(2):e12488. Epub 2018/06/30. doi: 10.1111/micc.12488. PubMed PMID: 29956866.
4. Liu K, Kang M, Li J, Qin W, Wang R. Prognostic value of the mRNA expression of members of the HSP90 family in non-small cell lung cancer. Exp Ther Med. 2019;17(4):2657-65. Epub 2019/04/02. doi: 10.3892/etm.2019.7228. PubMed PMID: 30930968; PubMed Central PMCID: PMCPMC6425268.
5. Barrett T, Wilhite SE, Ledoux P, Evangelista C, Kim IF, Tomashevsky M, et al. NCBI GEO: archive for functional genomics data sets--update. Nucleic Acids Res. 2013;41(Database issue):D991-5. Epub 2012/11/30. doi: 10.1093/nar/gks1193. PubMed PMID: 23193258; PubMed Central PMCID: PMCPMC3531084.
6. Gustavsson EK, Zhang D, Reynolds RH, Garcia-Ruiz S, Ryten M. ggtranscript: an R package for the visualization and interpretation of transcript isoforms using ggplot2. Bioinformatics. 2022;38(15):3844-6. Epub 2022/06/26. doi: 10.1093/bioinformatics/btac409. PubMed PMID: 35751589; PubMed Central PMCID: PMCPMC9344834.
7. Subramanian A, Tamayo P, Mootha VK, Mukherjee S, Ebert BL, Gillette MA, et al. Gene set enrichment analysis: a knowledge-based approach for interpreting genome-wide expression profiles. Proc Natl Acad Sci U S A. 2005;102(43):15545-50. Epub 2005/10/04. doi: 10.1073/pnas.0506580102. PubMed PMID: 16199517; PubMed Central PMCID: PMCPMC1239896.
8. Yu G, Wang LG, Han Y, He QY. clusterProfiler: an R package for comparing biological themes among gene clusters. Omics. 2012;16(5):284-7. Epub 2012/03/30. doi: 10.1089/omi.2011.0118.

PubMed PMID: 22455463; PubMed Central PMCID: PMCPMC3339379.
9. Li L, Lei Q, Zhang S, Kong L, Qin B. Screening and identification of key biomarkers in hepatocellular carcinoma: Evidence from bioinformatic analysis. Oncol Rep. 2017;38(5):2607-18. Epub 2017/09/14. doi: 10.3892/or.2017.5946. PubMed PMID: 28901457; PubMed Central PMCID: PMCPMC5780015.
10. Szklarczyk D, Gable AL, Nastou KC, Lyon D, Kirsch R, Pyysalo S, et al. The STRING database in 2021: customizable protein-protein networks, and functional characterization of user-uploaded gene/measurement sets. Nucleic Acids Res. 2021;49(D1):D605-d12. Epub 2020/11/26. doi: 10.1093/nar/gkaa1074. PubMed PMID: 33237311; PubMed Central PMCID: PMCPMC7779004.
11. Doncheva NT, Morris JH, Gorodkin J, Jensen LJ. Cytoscape StringApp: Network Analysis and Visualization of Proteomics Data. J Proteome Res. 2019;18(2):623-32. Epub 2018/11/20. doi: 10.1021/acs.jproteome.8b00702. PubMed PMID: 30450911; PubMed Central PMCID: PMCPMC6800166.
12. Tisherman SA, Schmicker RH, Brasel KJ, Bulger EM, Kerby JD, Minei JP, et al. Detailed description of all deaths in both the shock and traumatic brain injury hypertonic saline trials of the Resuscitation Outcomes Consortium. Ann Surg. 2015;261(3):586-90. Epub 2014/07/30. doi: 10.1097/sla.0000000000000837. PubMed PMID: 25072443; PubMed Central PMCID: PMCPMC4309746.
13. Eastridge BJ, Mabry RL, Seguin P, Cantrell J, Tops T, Uribe P, et al. Death on the battlefield (2001-2011): implications for the future of combat casualty care. J Trauma Acute Care Surg. 2012;73(6 Suppl 5):S431-7. Epub 2012/12/05. doi: 10.1097/TA.0b013e3182755dcc. PubMed PMID: 23192066.
14. Fox EE, Holcomb JB, Wade CE, Bulger EM, Tilley BC. Earlier Endpoints are Required for Hemorrhagic Shock Trials Among Severely Injured Patients. Shock. 2017;47(5):567-73. Epub 2017/02/17. doi: 10.1097/shk.0000000000000788. PubMed PMID: 28207628; PubMed Central PMCID: PMCPMC5392160.
15. Moore HB, Moore EE, Chapman MP, McVaney K, Bryskiewicz G, Blechar R, et al. Plasma-first resuscitation to treat haemorrhagic shock during emergency ground transportation in an urban area: a randomised trial. Lancet. 2018;392(10144):283-91. Epub 2018/07/24. doi: 10.1016/s0140-6736(18)31553-8. PubMed PMID: 30032977; PubMed Central PMCID: PMCPMC6284829.
16. Sperry JL, Guyette FX, Brown JB, Yazer MH, Triulzi DJ, Early-Young BJ, et al. Prehospital Plasma during Air Medical Transport in Trauma Patients at Risk for Hemorrhagic Shock. N Engl J Med. 2018;379(4):315-26. Epub 2018/07/26. doi: 10.1056/NEJMoa1802345. PubMed PMID: 30044935.
17. Kalkwarf KJ, Drake SA, Yang Y, Thetford C, Myers L, Brock M, et al. Bleeding to death in a big city: An analysis of all trauma deaths from hemorrhage in a metropolitan area during 1 year. J Trauma Acute Care Surg. 2020;89(4):716-22. Epub 2020/06/27. doi: 10.1097/ta.0000000000002833. PubMed PMID: 32590562.
18. Moore HB, Moore EE, Gonzalez E, Chapman MP, Chin TL, Silliman CC, et al. Hyperfibrinolysis, physiologic fibrinolysis, and fibrinolysis shutdown: the spectrum of postinjury fibrinolysis and relevance to antifibrinolytic therapy. J Trauma Acute Care Surg. 2014;77(6):811-7; discussion 7. Epub 2014/07/23. doi: 10.1097/ta.0000000000000341. PubMed PMID: 25051384; PubMed Central PMCID: PMCPMC4370273.
19. Simmons JW, Powell MF. Acute traumatic coagulopathy: pathophysiology and resuscitation. British Journal of Anaesthesia. 2016;117:iii31-iii43. doi: 10.1093/bja/aew328.

20. Brohi K, Gruen RL, Holcomb JB. Why are bleeding trauma patients still dying? Intensive Care Med. 2019;45(5):709-11. Epub 2019/02/12. doi: 10.1007/s00134-019-05560-x. PubMed PMID: 30741331.
21. Hoffman M, Pawlinski R. Hemostasis: old system, new players, new directions. Thromb Res. 2014;133 Suppl 1:S1-2. Epub 2014/04/25. doi: 10.1016/j.thromres.2014.03.001. PubMed PMID: 24759130.
22. Ivashkiv LB, Donlin LT. Regulation of type I interferon responses. Nat Rev Immunol. 2014;14(1):36-49. Epub 2013/12/24. doi: 10.1038/nri3581. PubMed PMID: 24362405; PubMed Central PMCID: PMCPMC4084561.
23. Seo JY, Yaneva R, Cresswell P. Viperin: a multifunctional, interferon-inducible protein that regulates virus replication. Cell Host Microbe. 2011;10(6):534-9. Epub 2011/12/20. doi: 10.1016/j.chom.2011.11.004. PubMed PMID: 22177558; PubMed Central PMCID: PMCPMC3246677.
24. Kurokawa C, Iankov ID, Galanis E. A key anti-viral protein, RSAD2/VIPERIN, restricts the release of measles virus from infected cells. Virus Res. 2019;263:145-50. Epub 2019/01/27. doi: 10.1016/j.virusres.2019.01.014. PubMed PMID: 30684519; PubMed Central PMCID: PMCPMC6615567.
25. Zhu H, Zheng J, Zhou Y, Wu T, Zhu T. Knockdown of RSAD2 attenuates B cell hyperactivity in patients with primary Sjögren's syndrome (pSS) via suppressing NF-κb signaling pathway. Mol Cell Biochem. 2021;476(5):2029-37. Epub 2021/01/30. doi: 10.1007/s11010-021-04070-z. PubMed PMID: 33512636.
26. Zhou X, Michal JJ, Zhang L, Ding B, Lunney JK, Liu B, et al. Interferon induced IFIT family genes in host antiviral defense. Int J Biol Sci. 2013;9(2):200-8. Epub 2013/03/06. doi: 10.7150/ijbs.5613. PubMed PMID: 23459883; PubMed Central PMCID: PMCPMC3584916.
27. Mehta P, McAuley DF, Brown M, Sanchez E, Tattersall RS, Manson JJ. COVID-19: consider cytokine storm syndromes and immunosuppression. Lancet. 2020;395(10229):1033-4. Epub 2020/03/21. doi: 10.1016/s0140-6736(20)30628-0. PubMed PMID: 32192578; PubMed Central PMCID: PMCPMC7270045.
28. Ahmad F, Kannan M, Ansari AW. Role of SARS-CoV-2 -induced cytokines and growth factors in coagulopathy and thromboembolism. Cytokine Growth Factor Rev. 2022;63:58-68. Epub 2021/11/10. doi: 10.1016/j.cytogfr.2021.10.007. PubMed PMID: 34750061; PubMed Central PMCID: PMCPMC8541834.
29. Imaizumi T, Hashimoto S, Sato R, Umetsu H, Aizawa T, Watanabe S, et al. IFIT Proteins Are Involved in CXCL10 Expression in Human Glomerular Endothelial Cells Treated with a Toll-Like Receptor 3 Agonist. Kidney Blood Press Res. 2021;46(1):74-83. Epub 2020/12/17. doi: 10.1159/000511915. PubMed PMID: 33326977.
30. Leisching G, Wiid I, Baker B. OAS1, 2, and 3: Significance During Active Tuberculosis? J Infect Dis. 2018;217(10):1517-21. Epub 2018/02/17. doi: 10.1093/infdis/jiy084. PubMed PMID: 29452387.
31. Fagone P, Nunnari G, Lazzara F, Longo A, Cambria D, Distefano G, et al. Induction of OAS gene family in HIV monocyte infected patients with high and low viral load. Antiviral Res. 2016;131:66-73. Epub 2016/04/25. doi: 10.1016/j.antiviral.2016.04.009. PubMed PMID: 27107898.
32. McDowell IC, Modak TH, Lane CE, Gomez-Chiarri M. Multi-species protein similarity clustering reveals novel expanded immune gene families in the eastern oyster Crassostrea virginica. Fish Shellfish Immunol. 2016;53:13-23. Epub 2016/04/02. doi: 10.1016/j.fsi.2016.03.157. PubMed

PMID: 27033806.
33. Miyashita M, Oshiumi H, Matsumoto M, Seya T. DDX60, a DEXD/H box helicase, is a novel antiviral factor promoting RIG-I-like receptor-mediated signaling. Mol Cell Biol. 2011;31(18):3802-19. Epub 2011/07/28. doi: 10.1128/mcb.01368-10. PubMed PMID: 21791617; PubMed Central PMCID: PMCPMC3165724.
34. Onomoto K, Jogi M, Yoo JS, Narita R, Morimoto S, Takemura A, et al. Critical role of an antiviral stress granule containing RIG-I and PKR in viral detection and innate immunity. PLoS One. 2012;7(8):e43031. Epub 2012/08/23. doi: 10.1371/journal.pone.0043031. PubMed PMID: 22912779; PubMed Central PMCID: PMCPMC3418241 study was partly funded by Nippon Boehringer Ingelheim. There are no patents, products in development or marketed products to declare. This does not alter the authors' adherence to all the PLoS ONE policies on sharing data and materials, as detailed online in the guide for authors.
35. Mathieu NA, Paparisto E, Barr SD, Spratt DE. HERC5 and the ISGylation Pathway: Critical Modulators of the Antiviral Immune Response. Viruses. 2021;13(6). Epub 2021/07/03. doi: 10.3390/v13061102. PubMed PMID: 34207696; PubMed Central PMCID: PMCPMC8228270.
36. Paparisto E, Woods MW, Coleman MD, Moghadasi SA, Kochar DS, Tom SK, et al. Evolution-Guided Structural and Functional Analyses of the HERC Family Reveal an Ancient Marine Origin and Determinants of Antiviral Activity. J Virol. 2018;92(13). Epub 2018/04/20. doi: 10.1128/jvi.00528-18. PubMed PMID: 29669830; PubMed Central PMCID: PMCPMC6002735.
37. Woods MW, Tong JG, Tom SK, Szabo PA, Cavanagh PC, Dikeakos JD, et al. Interferon-induced HERC5 is evolving under positive selection and inhibits HIV-1 particle production by a novel mechanism targeting Rev/RRE-dependent RNA nuclear export. Retrovirology. 2014;11:27. Epub 2014/04/04. doi: 10.1186/1742-4690-11-27. PubMed PMID: 24693865; PubMed Central PMCID: PMCPMC4021598.
