# Supplementary material for: Unveiling hub genes and biological pathways: A bioinformatics analysis of Trauma-Induced Coagulopathy (TIC)
Source: PLoS One. 2025 Apr 29;20(4):e0322043. doi: 10.1371/journal.pone.0322043 (PMC12040245; doi:10.1371/journal.pone.0322043)
Supplement: Attachment — Submitted filename: Response to Reviewers.docx [file pone.0322043.s002.docx]

**Response to Editor:**

Thank you for your detailed feedback and for providing clear guidance on the revisions required. We have addressed each point as follows:

1. Manuscript Style and File Naming:

We have carefully reviewed the PLOS ONE style requirements and have made the necessary adjustments to ensure that our manuscript fully complies with the formatting guidelines. Specifically:

The manuscript structure, headings, figure and table placements, and references have been revised following the sample templates provided (Main Body and Title/Authors).

File names have been updated to follow the journal’s naming conventions.

We believe the revised files now meet the journal's requirements, but we are happy to make further changes if needed.

2. Authors' Names in English:

Thank you for your clarification regarding author names. Since all authors are from China, we use Chinese pinyin for our names both in the manuscript and the online submission form.

3. Role of Funders Statement:

In the original manuscript, we acknowledged the following funding source:

“2023 Yuncheng City Basic Research Program (Free Exploration Category) Projects, Item No: YCKJ-2023052.”

The funders had no role in study design, data collection and analysis, decision to publish, or preparation of the manuscript.

This updated statement has been included in the cover letter accompanying this revision.

4. Reference List Review:

We have conducted a thorough review of the reference list to ensure it is complete, accurate, and up to date.

We have verified that all references cited are relevant and properly formatted according to the PLOS ONE guidelines.

5. DOI: dx.doi.org/10.17504/protocols.io.4r3l29bz4v1y/v1 (Private link for reviewers: https://www.protocols.io/private/77D8E398DB6C11EF88C30A58A9FEAC02 to be removed before publication.)

We hope that these revisions adequately address the concerns raised, and we are happy to make any additional changes if required. Thank you for your guidance and support throughout the review process.

**Response to Reviewer:**

Thank you for your detailed and constructive comments on our manuscript. We have carefully addressed each of your concerns and revised the manuscript accordingly. Below are our responses and the specific changes made:

1. Clarification of "GO" and "KEGG" Abbreviations:

We have spelled out "Gene Ontology (GO)" and "Kyoto Encyclopedia of Genes and Genomes (KEGG)" in the Introduction section (page 10), as suggested. This ensures readers are introduced to these terms earlier in the manuscript.

2. Explanation of "ROC":

We have briefly explained the term "Receiver Operating Characteristic (ROC)" in the Introduction to provide context for its use in the manuscript.

3. Subheadings for Methods Section:

Subheadings with appropriate numbering have been added to the Methods section (page 11). This improvement enhances the organization and readability of the section.

4. Introduction of Known Genes in TIC Pathogenesis:

We have expanded the Introduction to include relevant literature data on the role of specific known genes in the pathogenesis of TIC. This addition provides a stronger foundation for our study's context.

5. Revised Sentence on Page 14, Line 5:

The unclear sentence on page 14, line 5, has been revised for clarity and now includes the missing verb. The revised sentence ensures accurate and concise communication of the intended meaning.

6. Consistent Formatting of Gene Names:

Gene names have been formatted consistently in italics throughout the manuscript to comply with scientific conventions.

7. Toning Down Conclusions:

In the Conclusions section (page 20), we have revised the statement about our work being a "groundbreaking discovery." The new wording provides a more balanced conclusion.

8. Labeling of Axes in Figures:

Axes in Figures 3A, 3B, and 4A have been labeled to improve clarity and enhance the interpretability of the data.

9. Explanation of Tools in the Introduction:

Brief explanations of the tools "CYTOHubba," "MCODE," and "CTD" have been added to the Introduction. These descriptions provide readers with the necessary context for understanding the methods used in our study.

10. Table of 10 DEGs:

A new table summarizing the 10 differentially expressed genes (DEGs) identified in this study and their potential effects on TIC has been included. This table provides a clear and concise overview of these genes and their relevance to the study.

We have ensured that all revisions are highlighted in the revised manuscript for easy identification. Additionally, all formatting issues have been reviewed and corrected. We sincerely thank you for your constructive feedback, which has significantly improved the quality of our manuscript.

If there are any additional concerns or points that require clarification, we would be happy to address them.

Sincerely,

Lingang Zhang

January 26, 2025
